# Quantifying Limits on Replication, Death, and Quiescence of *Mycobacterium tuberculosis* in Mice

**DOI:** 10.3389/fmicb.2016.00862

**Published:** 2016-06-14

**Authors:** Margaret M. McDaniel, Nitin Krishna, Winode G. Handagama, Shigetoshi Eda, Vitaly V. Ganusov

**Affiliations:** ^1^National Institute for Mathematical and Biological SynthesisKnoxville, TN, USA; ^2^Department of Biochemistry, Cellular and Molecular Biology, University of TennesseeKnoxville, TN, USA; ^3^Department of Mathematics, University of TennesseeKnoxville, TN, USA; ^4^The College at the University of ChicagoChicago, IL, USA; ^5^Departments of Chemistry and Mathematics, Maryville CollegeMaryville, TN, USA; ^6^Department of Forestry, Wildlife and Fisheries, University of TennesseeKnoxville, TN, USA; ^7^Department of Microbiology, University of TennesseeKnoxville, TN, USA

**Keywords:** *Mycobacterium tuberculosis*, mathematical model, chronic infection, plasmid loss, pathogenesis, replication rate, death rate, mouse

## Abstract

When an individual is exposed to *Mycobacterium tuberculosis* (Mtb) three outcomes are possible: bacterial clearance, active disease, or latent infection. It is generally believed that most individuals exposed to Mtb become latently infected and carry the mycobacteria for life. How Mtb is maintained during this latent infection remains largely unknown. During an Mtb infection in mice, there is a phase of rapid increase in bacterial numbers in the murine lungs within the first 3 weeks, and then bacterial numbers either stabilize or increase slowly over the period of many months. It has been debated whether the relatively constant numbers of bacteria in the chronic infection result from latent (dormant, quiescent), non-replicating bacteria, or whether the observed Mtb cell numbers are due to balance between rapid replication and death. A recent study of mice, infected with a Mtb strain carrying an unstable plasmid, showed that during the chronic phase, Mtb was replicating at significant rates. Using experimental data from this study and mathematical modeling we investigated the limits of the rates of bacterial replication, death, and quiescence during Mtb infection of mice. First, we found that to explain the data the rates of bacterial replication and death could not be constant and had to decrease with time since infection unless there were large changes in plasmid segregation probability over time. While a decrease in the rate of Mtb replication with time since infection was expected due to depletion of host's resources, a decrease in the Mtb death rate was counterintuitive since Mtb-specific immune response, appearing in the lungs 3–4 weeks after infection, should increase removal of bacteria. Interestingly, we found no significant correlation between estimated rates of Mtb replication and death suggesting the decline in these rates was driven by independent mechanisms. Second, we found that the data could not be explained by assuming that bacteria do not die, suggesting that some removal of bacteria from lungs of these mice had to occur even though the total bacterial counts in these mice always increased over time. Third and finally, we showed that to explain the data the majority of bacterial cells (at least ~60%) must be replicating in the chronic phase of infection further challenging widespread belief of nonreplicating Mtb in latency. Our predictions were robust to some changes in the structure of the model, for example, when the loss of plasmid-bearing cells was mainly due to high fitness cost of the plasmid. Further studies should determine if more mechanistic models for Mtb dynamics are also able to accurately explain these data.

## 1. Introduction

Tuberculosis (TB) is one of the oldest known human infectious diseases and together with HIV/AIDS is now the greatest global killer due to a single infectious agent (WHO, 2013, 2014). Roughly 9 million new active tuberculosis cases are diagnosed and over 1 million die from the disease each year (Bourzac, 2014; Murray et al., 2014; WHO, 2014; Glaziou et al., 2015). Throughout human history, TB is estimated to have killed over 1 billion individuals (Paulson, 2013).

TB is caused by *Mycobacterium tuberculosis* (Mtb), an actinomycete closely related to saprophytic bacteria. Natural infection with Mtb occurs by inhalation, followed by ingestion of bacteria by lung-resident alveolar macrophages that provide the major initial replication niche for the pathogen (Repasy et al., 2013). After phagocytosis by the macrophage, the mycobacterium is retained within a phagocytic vacuole (phagosome) by inhibition of phagosome-lysosome fusion (Takayama et al., 2005).

Following exposure of humans to Mtb, three outcomes are possible (Flynn and Chan, 2001; Monack et al., 2004; Gideon and Flynn, 2011). First, bacteria may get rapidly engulfed by macrophages or other cells in the lung and cleared. Second, initial innate response may fail clearing the bacteria, and the bacterial population will grow in size leading to active disease. Third, initially replicating bacteria may be contained by the adaptive immunity within structures called granulomas leading to latent infection in which bacteria persist for long periods of time, often the lifespan of the host (Flynn and Chan, 2001; Monack et al., 2004; Gideon and Flynn, 2011). The relative proportion of exposed individuals resulting in particular type of infection is generally poorly understood, but some studies cite 5%/95% ratio for active disease vs. latent infection, with few if any individuals clearing Mtb completely (Kamat et al., 1966; Comstock et al., 1974; O'Garra et al., 2013; Getahun et al., 2015). A nearly 20 year old extrapolation suggests that roughly one-third of the human population is latently infected (Dye et al., 1999), although this estimate needs to be re-evaluated given novel methods of detecting latent infection and overall decline in TB incidence (Getahun et al., 2015). Furthermore, it is generally believed that latently infected individuals have ~5–10% life-time chance of developing active disease (Monack et al., 2004; Barry et al., 2009; Horsburgh et al., 2010; Shea et al., 2014; Zheng et al., 2014). The most recent estimates for TB reactivation have been questioned, however, due to biases in selecting patients and some assumptions involved in calculating reactivation rates (Houben et al., 2014; Sanderson et al., 2014). Irrespectively of the exact rate of reactivation, the majority of reactivation cases appear to be contracted in the developing world and are strongly associated with overcrowding and malnutrition (Lawn and Zumla, 2011).

While some individuals exposed to Mtb do become latently infected, it remains poorly understood how bacteria are maintained during the latent infection in humans. The location and physiological state of the bacilli in latent infection remain largely unknown and has hindered efforts to combat reactivation of latent tuberculosis. Generally there are two possibilities of how Mtb is able to persist during latent infection. First, the bacteria may enter a non-replicative, dormant stage hidden from the host immunity. Second, the bacteria may be maintained by the constant replication and removal by immunity. A combination of these two mechanisms where a fraction of bacteria actively replicates and another fraction is quiescent, is also possible. Distinguishing between these alternative mechanisms is potentially difficult in humans but can be addressed by using Mtb infection of laboratory animals.

Mtb is capable of infecting multiple mammalian species, and symptoms and overall pathogenesis caused by Mtb vary from species to species (O'Toole, 2010; Franzblau et al., 2012; Dartois and Barry, 2013; Myllymäki et al., 2015). Due to low cost, ease of genetic manipulation, and availability of reagents mice are the most commonly used experimental model of Mtb infection and immune response dynamics, despite major differences in pathogenesis of TB in humans and mice (Dartois and Barry, 2013; Myllymäki et al., 2015). In mice infected intranasally with a low dose of Mtb, the total number of bacteria in the lungs increases exponentially for 3 weeks and then plateaus for several months (Gill et al., 2009; Ernst, 2012) (but a decline in Mtb counts after the peak was observed in some studies Gallegos et al., 2008; Zhang et al., 2012). As in the case of human infection, how Mtb is maintained in the chronic phase of infection of mice is also poorly understood. Because this state of apparent latency (also called dormancy or quiescence) may make bacteria resistant to host immunity or antibiotics, understanding mechanisms of Mtb maintenance during chronic infection is of high importance. One commonly accepted view is that bacilli, residing in granulomas, are responsible for disease reactivation and that these bacilli become dormant in response to high-stress conditions (Barry et al., 2009). Although the exact location of viable dormant mycobacteria during persistent infections has yet to be elucidated, bacteria are often found inside macrophages within granulomas, which are formed in response to persistent intracellular pathogens (Monack et al., 2004).

In contrast with the “latency/dormancy” hypothesis, several other studies suggested that there may be substantial continuous bacterial replication during the chronic stages of infection (Boshoff and Barry, 2005; Gill et al., 2009). Past studies of mouse lung histopathology during chronic infection have indicated deterioration of tissue health and progressive inflammation, despite stable bacterial counts (Rhoades et al., 1997; Boshoff and Barry, 2005). These results are inconsistent with non-replicating bacteria, and show evidence that substantial replication and killing of mycobacteria may still be taking place during chronic stages of infection, even though bacterial population counts remain stable, which can be referred to as “dynamic equilibrium.”

An important breakthrough in understanding chronic Mtb persistence (in mice) was achieved recently (Gill et al., 2009). The authors used a Mtb strain carrying a plasmid pBP10 with kanamycin resistance. The plasmid was stably maintained in culture in the presence of kanamycin but was lost in the absence of antibiotic at a constant (per cell division) rate (Bachrach et al., 2000; Gill et al., 2009). Because the frequency of plasmid-bearing cells in the population declines only when there is cell division, the dynamics of bacteria can be used to estimate the rate of Mtb replication *in vivo* (Gill et al., 2009). By combining the use of a simple mathematical model and experimental data on the dynamics of plasmid-bearing and plasmid-free mycobacteria the authors for the first time provided quantitative estimates of how quickly Mtb replicated and died during infection of mice. The analysis revealed that in comparison to acute infection (first 2 weeks) bacterial replication rate was reduced only four fold during the chronic infection providing strong support for the “dynamic equilibrium” hypothesis.

Here we use mathematical modeling to reanalyze these previously published experimental data and to investigate robustness of previously found estimates of the rates of Mtb replication and death during infection in mice. Our analysis suggests that estimates of Mtb replication and death rates strongly depend on the model structure and value of other parameters, although some conclusions such as decline in the rate of Mtb replication and death over the course of infection appear to be relatively robust assuming a constancy of plasmid loss probability. Our work also highlights the need for additional independent measurements of the plasmid segregation probability and plasmid fitness cost *in vivo* to better quantify Mtb dynamics, e.g., to estimate the fraction of quiescent, nonreplicating bacteria.

## 2. Materials and methods

### 2.1. Data

**Figure 1:**
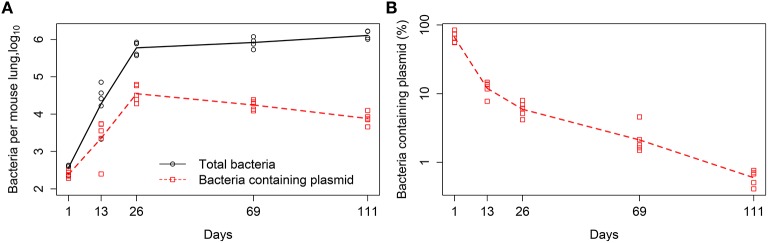
**Kinetics of Mtb replication in mice (Gill et al., 2009)**. Mice were infected intranasally with a low dose of Mtb strain H37Rv carrying a plasmid pBP10; the plasmid was lost by bacteria at cell division. Data are for the total number of bacteria and number of plasmid-bearing bacteria in the murine lungs **(A)** or the percent of plasmid-bearing cells in the population **(B)**. Markers denote measurements for individual mice (*n* = 5 per time point) and lines are connecting geometric means of bacterial counts. The total deviation of the log_10_-transformed data from the geometric mean in **(A)** is 3.26, and therefore according to the lack of fit test, best fit models should have the sum of squared residuals close to this value (Bates and Watts, 1988).

In our analysis we used experimental data from previously published study (Gill et al., 2009), in which the details of experimental procedures are described in detail. In short, C57BL/6 mice were aerosol infected with a low dose of Mtb strain H37Rv carrying a plasmid pBP10. The plasmid is thought to be a low copy number plasmid although the exact average number of plasmid copies per Mtb cell has not been published (Bachrach et al., 2000). Mtb cell numbers in the lungs were measured at 5 different time points post infection (day 1, 13, 26, 69, 111) by plating lung homogenates from *n* = 5 mice per time point and counting the number of colony forming units (CFUs). The total CFUs per lung and the percent of plasmid-bearing cells were recorded (Figure 1). Following previous work (Gill et al., 2009), we define time intervals during which parameters of the models (see below) are assumed to be constant; these intervals are days 1–13, 13–26, 26–69, and 69–111. We also provide these experimental data in Supplemental Information (Data Sheet 1).

### 2.2. Main mathematical models

#### 2.2.1. Extended model

In order to quantify the rate of Mtb replication, *r*(*t*), and death, δ(*t*), *in vivo*, Gill et al. (2009) proposed a mathematical model that described the change in the number of plasmid-carrying and plasmid-free bacteria with respect to time. Plasmid-free and plasmid-bearing strains were assumed to have the same growth and death rates, which were taken to be constant within time intervals but could change between time intervals (Gill et al., 2009, Figure 2). We extended the model by also allowing the segregation probability *s* = *s*(*t*) to change with time but be constant for a specific time interval. Under these assumptions, the extended model for Mtb replication in mice becomes:

(1) $$\begin{array}{l} \frac{dP\left(t\right)}{dt}=\left[r\left(t\right)\left(1-s\left(t\right)\right)-\delta \left(t\right)\right]P\left(t\right), \end{array}$$

(2) $$\begin{array}{l} \frac{dF\left(t\right)}{dt}=\left[r\left(t\right)-\delta \left(t\right)\right]F\left(t\right)+r\left(t\right)s\left(t\right)P\left(t\right), \end{array}$$

(3) $$\begin{array}{l} \frac{dN\left(t\right)}{dt}=\left[r\left(t\right)-\delta \left(t\right)\right]N\left(t\right), \end{array}$$

where *N*(*t*) = *P*(*t*) + *F*(*t*) is the total number of bacteria, *P*(*t*) is the number of plasmid-carrying bacteria, *F*(*t*) is the number of plasmid-free bacteria, and *t* is time in days. In the model, the segregation probability *s*(*t*) denotes the probability that a plasmid-free cell will be produced from a division of a plasmid-bearing cell. Parameters for the mathematical model are defined as follows:

(4) $$\begin{array}{l} r\left(t\right),\delta \left(t\right),s\left(t\right)=\left\{\begin{array}{cc} \left(r_{1},\delta_{1},s_{1}\right), & if1\leq t<13,\\ \left(r_{2},\delta_{2},s_{2}\right), & if13\leq t<26,\\ \left(r_{3},\delta_{3},s_{3}\right), & if26\leq t<69,\\ \left(r_{4},\delta_{4},s_{4}\right), & if69\leq t<111. \end{array}\right\} \end{array}$$

**Figure 2:**
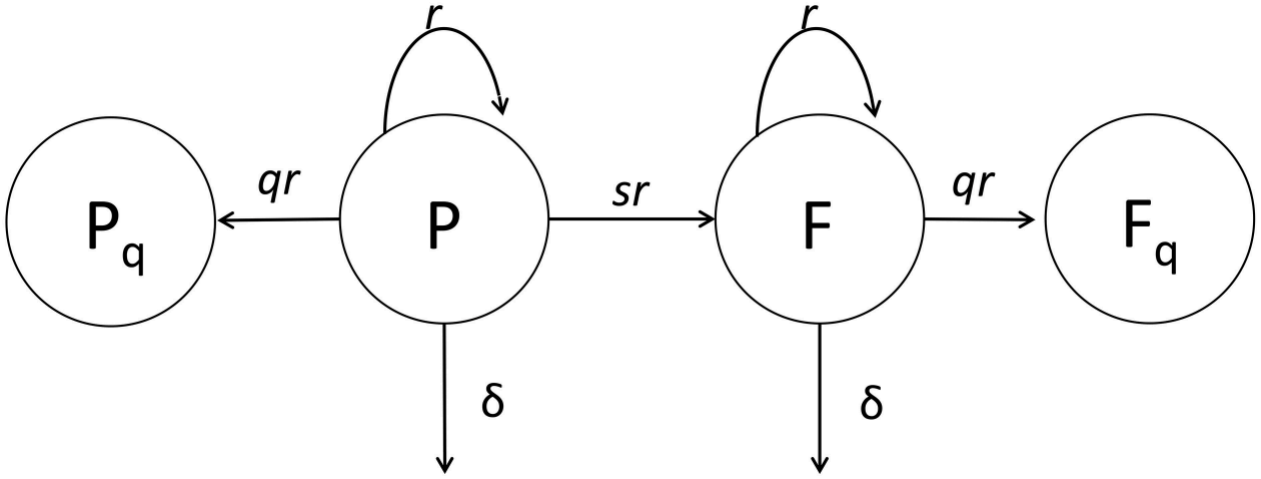
**Cartoon of the general mathematical model of Mtb dynamics in mice**. In the model, plasmid-bearing (*P*) and plasmid-free (*F*) bacteria replicate and die at rates *r* and δ, respectively, and plasmid-free bacteria are formed during a division of plasmid-carrying cell with the probability *s*. We consider two versions of the general model. In our first version (*extended* model) *q* = 0 and rates *r* and δ and probability *s* could all be time-dependent (see Equations 1 and 2) while the original study of Gill et al. (2009) had a constant segregation probability *s*. In the second version (*quiescence* model) we allow formation of quiescent cells, *P*_*q*_ and *F*_*q*_, following cell division with a probability *q* > 0 (see Equations 7–10). In the quiescence model, the rates *r* and δ and probability *q* could be time-dependent, while probability of plasmid loss was fixed to a value determined from *in vitro* experiments, *s* = 0.18 (Gill et al., 2009).

Note that in the previous work the probability of plasmid segregation was determined *in vitro* and was fixed to value *s* = 0.18 in further analyses of *in vivo* data (Gill et al., 2009). However, in general segregation probability could vary from 0 to 1 with *s* = 1 representing a situation when following division of a plasmid-bearing cell one of the daughter cells is always plasmid-free.

#### 2.2.2. Linear regressions

In the extended model (Equations 1–3), for every time period in the data we have three parameters, *r*_*i*_, δ_*i*_, and *s*_*i*_ (see Equation 4). Given that for a given time period we only have measurements of two Mtb subpopulations (plasmid-bearing and plasmid-free cells), we can only estimate two slopes from experimental data (Gill et al., 2009, Figure 1A). Given two slopes, the following relationships between parameters exist

(5) $$\begin{array}{l} s_{i}r_{i}=SlopeN_{i}-SlopeP_{i}, \end{array}$$

(6) $$\begin{array}{l} r_{i}-\delta_{i}=SlopeN_{i}, \end{array}$$

where *SlopeN*_*i*_ and *SlopeP*_*i*_ are slopes estimated by fitting linear regression lines through plots of the *i*^th^ intervals of ln[*N*(*t*)] and ln[*P*(*t*)] vs. *t*, respectively (Gill et al., 2009). Because only two slopes are estimated from the data while the extended model has three parameters, we consider 3 alternative sub-models for Mtb dynamics: (i) model with a constant, time interval-independent segregation probability *s*, (ii) model with a constant, time interval-independent replication rate *r*, and (iii) model with a constant, time interval-independent death rate δ. With one of the parameters of the model being set to a fixed value, other parameters can be estimated from the data using linear regressions for log-transformed total number of bacteria (*N*(*t*)) and the number of plasmid-bearing cells (*P*(*t*)).

#### 2.2.3. Quiescence model

It is possible that not all bacteria in mice are replicating. To investigate how large the fraction of non-replicating bacteria could be, we propose the following mathematical model. In the model, following cell division a fraction of bacteria go into a non-replicating quiescent state at a time-dependent rate *q*(*t*). Assuming that the segregation probability is constant in this model, *s*(*t*) = *s*, and that both *q*(*t*) and *s* are small [so then product *sq*(*t*) can be neglected] the dynamics of plasmid-bearing and plasmid-free cells is given by equations:

(7) $$\begin{array}{l} \frac{dP\left(t\right)}{dt}=\left[r\left(t\right)\left(1-s-q\left(t\right)\right)-\delta \left(t\right)\right]P\left(t\right), \end{array}$$

(8) $$\begin{array}{l} \frac{dF\left(t\right)}{dt}=sr\left(t\right)P\left(t\right)+\left[r\left(t\right)\left(1-q\left(t\right)\right)-\delta \left(t\right)\right]F\left(t\right), \end{array}$$

(9) $$\begin{array}{l} \frac{dP_{q}\left(t\right)}{dt}=q\left(t\right)r\left(t\right)P\left(t\right), \end{array}$$

(10) $$\begin{array}{l} \frac{dF_{q}\left(t\right)}{dt}=q\left(t\right)r\left(t\right)F\left(t\right), \end{array}$$

where *P*_*q*_(*t*) and *F*_*q*_(*t*) are the total number of dormant (or quiescent) plasmid-carrying and plasmid-free bacteria, respectively. We consider two situations when dormant bacteria are able to revert their state and grow on plates *in vitro* (then the total number of bacteria *N*(*t*) = *P*(*t*) + *F*(*t*) + *P*_*q*_(*t*) + *F*_*q*_(*t*)), and when dormant bacteria are unable to replicate *in vitro* and *in vivo* (*N*(*t*) = *P*(*t*) + *F*(*t*)). Similar calculations apply to populations of plasmid-bearing and plasmid-free cells. In this model, the rates of bacterial replication and death and quiescence probability are defined as a piecewise constant function similarly to Equation (4). As another alternative model we also consider a model in which quiescent cells are removed at the same rate as replicating cells; results of the analysis of this model are given in Supplemental Information (Table S1).

### 2.3. Alternative mathematical models

#### 2.3.1. Model with fitness cost for the plasmid

Previous analysis assumed that the reason for accumulation of plasmid-free cells in the population was due to a high segregational probability *s* = 0.18 (Gill et al., 2009). This in part stemmed from the observation of a linear decline of the log-transformed fraction of plasmid-bearing cells in the population over time in *in vitro* experiments (Gill et al., 2009). However, because of noise in the data it is possible that both segregational probability and fitness cost of carrying the plasmid may contribute to the overall increase in the fraction of plasmid-free cells (Ganusov and Brilkov, 2002). To investigate this issue further we modified the extended mathematical model (Equations 1–3) by fixing *s*(*t*) = *s* and introducing fitness cost *c* in the bacterial replication rate (Gill et al., 2009)

(11) $$\begin{array}{l} \frac{dP\left(t\right)}{dt}=\left[r\left(t\right)\left(1-s\right)\left(1-c\right)-\delta \left(t\right)\right]P\left(t\right), \end{array}$$

(12) $$\begin{array}{l} \frac{dF\left(t\right)}{dt}=\left[r\left(t\right)-\delta \left(t\right)\right]F\left(t\right)+s\left(1-c\right)r\left(t\right)P\left(t\right). \end{array}$$

Using simple algebra it can be shown that change in the total number of bacterial cells (*N*(*t*)) and the frequency of plasmid-bearing cells in the population (*f*(*t*)) are given by equations

(13) $$\begin{array}{l} \frac{dN\left(t\right)}{dt}=r\left(t\right)N\left(t\right)\left(1-cf\left(t\right)\right), \end{array}$$

(14) $$\begin{array}{l} \frac{df\left(t\right)}{dt}=-r\left(t\right)f\left(t\right)\left[s\left(1-c\right)+c\left(1-f\left(t\right)\right)\right]. \end{array}$$

In this model the change in the total number of bacteria and the frequency of plasmid-bearing bacteria now depend on the frequency of plasmid-bearing cells in the population *f*(*t*). Importantly, for *f*(*t*) ≈ 1, the rate of decline of the log-transformed frequency of plasmid-bearing cells is now determined by both the segregational probability *s* and fitness cost *c*, and as previously, is directly proportional to the rate of Mtb replication *r*(*t*). By using previously published data from *in vitro* experiments on the decline in the frequency of plasmid-bearing cells we found that the data could be explained assuming a range of values for *s* and *c*; at the extreme case of *s* = 0.04 and *c* = 0.16 the model was still adequate at describing the data (Gill et al., 2009) as compared to a model with no fitness cost (*s* = 0.18 and *c* = 0) based on AIC (results not shown).

#### 2.3.2. Logistic growth model

Our main mathematical models did not make any assumptions of specific mechanisms regulating changes in the rate of Mtb replication and death. We therefore investigated if a model which assumes logistic growth for Mtb was able to accurately explain the experimental data. In this model bacteria replicate at a density-dependent rate with a maximum replication rate *r* and carrying capacity *K*:

(15) $$\begin{array}{l} \frac{dP\left(t\right)}{dt}=r\left(1-s\right)\left(1-\frac{P\left(t\right)+F\left(t\right)}{K}\right)P\left(t\right)-\delta P\left(t\right), \end{array}$$

(16) $$\begin{array}{l} \frac{dF\left(t\right)}{dt}=r\left(1-\frac{P\left(t\right)+F\left(t\right)}{K}\right)F\left(t\right)\\ +\;rs\left(1-\frac{P\left(t\right)+F\left(t\right)}{K}\right)P\left(t\right)-\delta F\left(t\right). \end{array}$$

In total, this model has 5 parameters: *r*, *K*, δ, *P*(0) and *F*(0) when *s* = 0.18.

### 2.4. Statistics

Some of our analyses focused on estimating replication and death rates of Mtb using linear regressions as outlined above. In addition, to investigate further how many parameters are needed to accurately explain the data we also fitted a series of mathematical models of different levels of complexity to the data which included the number of total and plasmid-bearing bacteria in murine lungs (Figure 1A). In these fits we log_10_-transformed model predictions and the data.

The models were fitted in R (version 3.1.0) using *modFit* routine in *FME* package by minimizing the sum of squared residuals. Numerical solutions to the system of equations were obtained using ODE solver *lsoda* (from the *deSolve* package) with an absolute and relative error tolerance of 10^−6^. The L-BFGS-B and Marquart algorithms were used to find parameter estimates providing the best fit of models to data. Discrimination between different models was done using corrected AIC (Burnham and Anderson, 2002). The model with the minimal AIC score among all tested models was viewed as the best fit model, but a difference of AIC score of 1–3 between best fit and second best fit models was generally viewed as not significant. A difference in AIC score of greater than 3 was generally viewed as significant, so the model with a lower AIC score was interpreted as a better fit model (Burnham and Anderson, 2002).

To test the statistical significance of the differences between the replication (and death) rates found for different time intervals using linear regressions with a fixed segregation probability *s* = 0.18, we used a bootstrap approach (Efron and Tibshirani, 1993). We resampled the data corresponding to individual mice with replacement to generate 5 measurements of total and plasmid-bearing bacteria per time point 10^4^ times using the *RandomChoice* routine in *Mathematica*. Values for growth and death rates for each interval were then calculated using Equations (5) and (6), and bootstrap samples were used to calculate the difference between estimates of growth or death rates for two consecutive time periods (e.g., *r*_2_ − *r*_1_). The *p*-values, indicating statistical significance of the difference, were calculated as number of bootstrapped negative differences divided by 10^4^. Confidence intervals for parameters of our best ODE-based models (e.g., Equations 1 and 2) were determined by resampling data for individual mice with replacement using 10^3^ simulations.

## 3. Results

In their pioneering study, Gill et al. (2009) infected mice with Mtb strain carrying a plasmid and followed the dynamics of the total number of bacteria and the number of plasmid-bearing bacteria (Figure 1). The authors developed a very simple mathematical model, based on few assumptions, and estimated the rates of Mtb replication and death from their experimental data. However, the original study did not fully address the question of how robust the obtained estimates were relative to the assumptions made in the model. Here we extend this study by determining robustness of parameter estimates relative to several changes in the model assumptions (Figure 2).

### Fewer parameters are needed to explain the data

In their original analysis, Gill et al. (2009) estimated four replication (*r*) and four death (δ) rates for different time intervals during Mtb infection of mice (see Section 2 for more detail). While replication rate was predicted to decline during the infection, death rate was changing non-monotonically with time since infection and was nonsignificantly different from zero in one time interval. To investigate whether these individual parameters truly change during the infection we used a bootstrap approach whereby we resampled our data with replacement to create bootstrap replicates and estimated *r* and δ for four time periods. Assuming a fixed segregation probability *s* = 0.18, we found that there were significant differences between the following parameter pairs: *r*_1_ and *r*_2_, *r*_2_ and *r*_3_, and δ_1_ and δ_2_ (Table 1). Thus, we show that during chronic phase of infection (more than 69 days post infection) there is little change in the rate of Mtb replication and death and that the rate of Mtb death was only changing during first 2 weeks of infection. To corroborate these findings we fitted a series of ODE-based mathematical models in which parameters for cell replication and death were kept constant for different time periods (Table 2). Model fitting revealed that indeed the best fit was provided by the model in which three replication and two death rates during the infection were needed (see “Constrained model 1” in Table 2). Fits predicted that both replication and death rates decline during the infection. Importantly, while the decline in the Mtb replication rate during infection can be explained (e.g., depletion of resources), decline of death rate is harder to explain especially because 3 weeks post infection, Mtb-specific T cell responses arrive to the lung, and are expected to increase removal rate of bacteria by activating lung macrophages (Jung et al., 2005).

**Table 1:** **Bootstrap analysis suggested non-significant changes in the rates of Mtb replication and death for several time periods**.

**Growth rate**	***p*-value**	**Death rate**	***p*-value**
*r*_1_, *r*_2_	< 10^−4^	δ_1_, δ_2_	< 10^−4^
*r*_2_, *r*_3_	0.0064	δ_2_, δ_3_	0.11
*r*_3_, *r*_4_	0.19	δ_3_, δ_4_	0.19

We resampled experimental data of Gill et al. (2009) 10^4^ times with replacement and generated distribution of estimates for replication (r) and death (δ) rates during four time periods assuming a fixed segregation probability s = 0.18. The indicated p-values are calculated from the distribution of differences between two consecutive rates.

**Table 2:** **Fitting the extended mathematical model to experimental data suggested that few parameters were needed to explain the data**.

Full Model	(day^−1^)	*r*_1_	*r*_3_	*r*_3_	*r*_4_	δ_1_	δ_2_	δ_3_	δ_4_	AIC	SSR
		0.78	0.30	0.13	0.17	0.46	0.04	0.13	0.16	−116.98	3.26
Constrained Model 1	(day^−1^)	*r*_1_	*r*_2_	*r*_3_		δ_1_	δ_2_			AIC	SSR
		0.72	0.40	0.14		0.40	0.13			−124.72	3.29
Constrained Model 2	(day^−1^)	*r*_1_	*r*_2_			δ_1_	δ_2_			AIC	SSR
		0.49	0.21			0.18	0.20			−114.45	4.24
Constrained Model 3	(day^−1^)	*r*_1_	*r*_2_			δ_1_				AIC	SSR
		0.50	0.20			0.19				−116.51	4.27

Assuming s = 0.18 we numerically solved the model given in Equations (1) and (2), fitted model solutions to the data on dynamics of the total number of bacteria and the number of plasmid-bearing bacteria (Figure 1A), and estimated model parameters (see Equation 4). Resulting parameters, the sum of squared residuals (SSR), and AIC scores for the model fits are indicated in columns. The lowest AIC score was provided by Constrained Model 1 (“3r∕2δ” model), with three replication (r_1_, r_2_, r_3_) and 2 death (δ_1_, δ_2_) rates which is similar to the results found in Table 1. This model could accurately describe experimental data based on the lack of fit test [F_(3, 40)_ = 0.11, p = 0.95], and was not worse than the full model with four replication and four death rates [F-test for nested models, F_(3, 40)_ = 0.11, p = 0.95]. Visually model fits were indistinguishable from lines shown in Figure 1A. For our minimal (“3r∕2δ”) model we have the following 95% confidence intervals obtained by bootstrapping the data with replacement 10^3^ times: r_1_ = 0.72 (0.63−0.82)/day, r_2_ = 0.40 (0.33−0.49)/day, r_3_ = 0.14 (0.12−0.15)/day, δ_1_ = 0.40 (0.32−0.49)/day, δ_2_ = 0.13 (0.11−0.14)/day, P(0) = 200 (165–240), F(0) = 67 (26–107).

### Varying plasmid segregation probability, replication and death rates

Prior *in vitro* experiments showed that the plasmid pBP10 has a constant probability loss per generation, *s* = 0.18 (Gill et al., 2009). Although the *in vitro* segregation probability was independent of the growth rate, it is unknown whether the plasmid segregation probability is different between *in vitro* and *in vivo*, and whether segregation probability changes during Mtb infection *in vivo*. Some experimental data do suggest that plasmid segregation rates may depend on growth conditions which are likely to change during *in vivo* infection (Wouters et al., 1980; Popova et al., 1992; Mosrati et al., 1993; Smith and Bidochka, 1998; Ganusov and Brilkov, 2002). For example, the number of plasmid copies per bacterial cell plays a major role in determining the probability of formation of a plasmid-free cell (Paulsson and Ehrenberg, 1998, 2000), and some studies suggest changes in plasmid copy number in different culture conditions (Jones et al., 1980; Lin-Chao and Bremer, 1986). Because pBP10 is a low copy number plasmid (Bachrach et al., 2000), even small changes in the copy number are likely to lead to a large change in the probability of plasmid loss.

**Figure 3:**
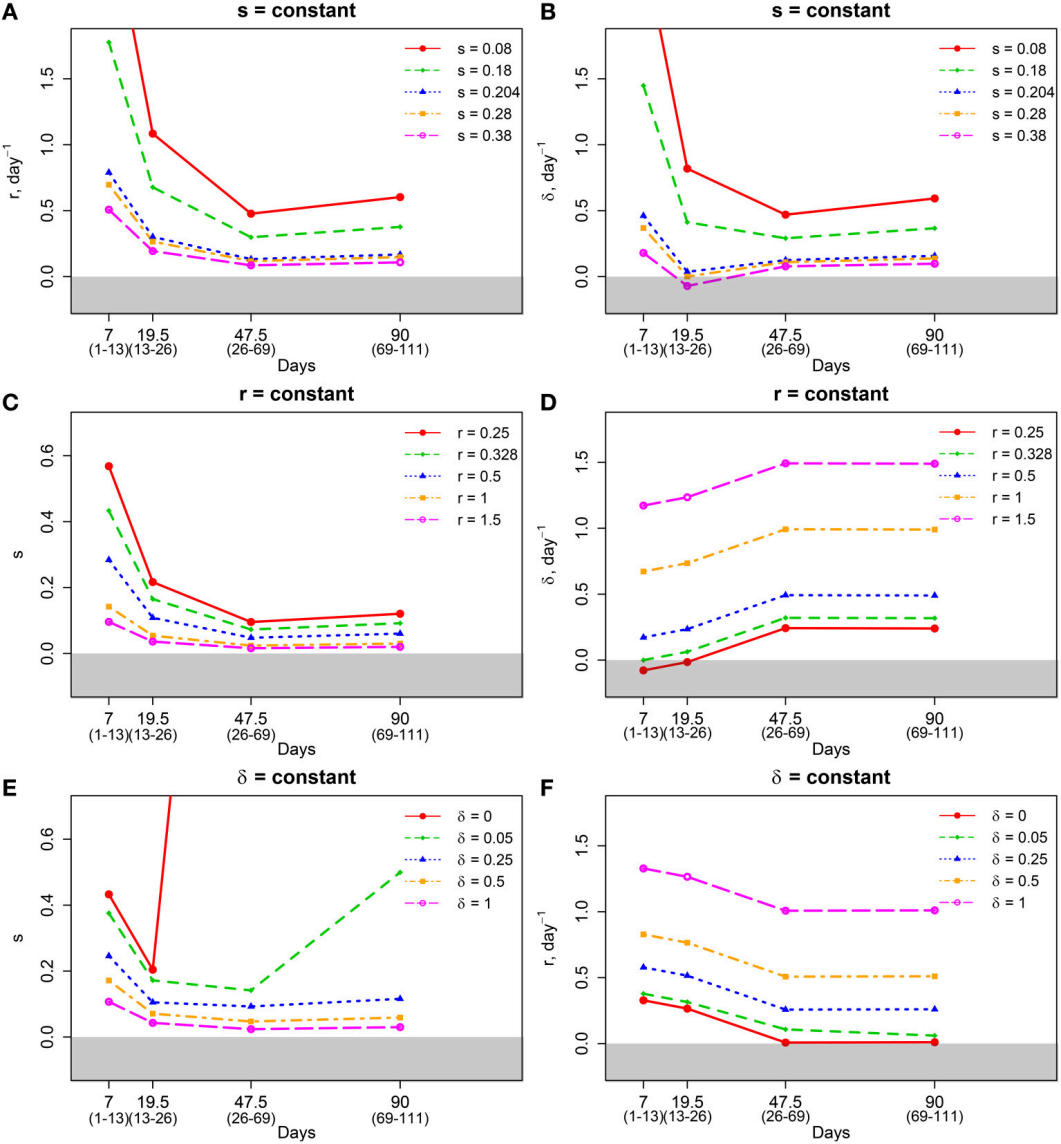
**Dramatic dependence of the estimated replication *r* and death δ rates and segregation probability *s* on the assumptions in the model**. We made assumptions on the value of a constant segregation probability *s* **(A,B)**, constant replication rate *r* **(C,D)**, and constant death rate δ **(E,F)** and estimated remaining parameters using linear regressions (Equations 5 and 6) from experimental data (Figure 1A). Gray areas of each plot denote values that are physiologically impossible. Plotted values were also listed in Tables 3–5.

Mathematically, the segregation probability of plasmids may vary between 0 and 1. A segregation probability of 1 implies that after a replication cycle, only one of daughter cells has the plasmid; a value greater than 1 is therefore biologically infeasible (unless the plasmid can be degraded intracellularly or “expelled” by a cell). Therefore, we fixed segregation probability *s* to different constant values and estimated Mtb replication and death rate using Equations (5) and (6). This analysis suggested that the rates of replication and death strongly depended on the actual value of the segregation probability, and that lower values of segregation probability naturally led to higher values of the Mtb replication and death rates (Table 3 and Figures 3A,B). Furthermore, analysis suggested that there was a limit on the constant segregation probability for the parameters to be all positive; this value is *s* = 0.204 and it is larger than the value found in *in vitro* experiments (Gill et al., 2009).

**Table 3:** **Estimates of Mtb replication and death rates strongly depended on the value for the plasmid segregation probability *s***.

***s***	**Day 1–13**		**Day 13–26**		**Day 26–69**		**Day 69–111**	
	***r*_1_ (day^−1^)**	**δ_1_ (day^−1^)**	***r*_2_ (day^−1^)**	**δ_2_ (day^−1^)**	***r*_3_ (day^−1^)**	**δ_3_ (day^−1^)**	***r*_4_ (day^−1^)**	**δ_4_ (day^−1^)**
0.05	2.842	2.514	1.084	0.819	0.477	0.470	0.603	0.592
0.08	1.776	1.448	0.678	0.412	0.298	0.290	0.377	0.366
0.18	0.789	0.461	0.301	0.036	0.133	0.125	0.167	0.157
0.204	0.697	0.368	0.266	0.000	0.117	0.109	0.148	0.137
0.28	0.508	0.179	0.194	−0.072	0.085	0.078	0.108	0.097
0.38	0.374	0.046	0.143	−0.123	0.063	0.055	0.079	0.069
0.48	0.296	−0.032	0.113	−0.152	0.050	0.042	0.063	0.052
*S_max_*		0.433		0.204		3.108		2.920

We fixed the value of s = s_i_ and calculated replication r_i_ and death δ_i_ rates using linear regressions in Equations (5) and (6). Gray boxes denote parameters outside physiologically feasible ranges. The value of segregation probability s yielding δ_i_ = 0 was the upper bound for s in that interval (s_max_). The smallest of these maximum values, s_max_ = 0.204, was the upper bound for the segregation probability when it was constant throughout infection.

Previous work also established that the rate of Mtb replication should decline during the Mtb infection of mice (Gill et al., 2009). We therefore investigated if the general model (Equations 1 and 2) in which the rate of Mtb replication is constant during the infection can explain the data for biologically reasonable parameters assuming that both death rate and segregation probability change with time since infection. Our analysis suggested that it is possible (Table 4). The analysis also revealed that there is a limit on the constant rate of Mtb replication, *r* = 0.328 per day below which the data cannot be explained (Table 4 and Figures 3C,D). Additionally, in order for the model with a constant replication rate to be consistent with the data, segregation probability has to change dramatically, 5–7 fold (e.g., for *r* = 1 per day, see Table 4) over the course of infection. Independent measurements of the plasmid segregation probability in these *in vivo* experiments would further allow to determine if a constant rate of Mtb replication is consistent with the data.

**Table 4:** **Replication rate of Mtb replication was unlikely to be constant during Mtb infection of mice**.

***r***	**Day 1–13**		**Day 13–26**		**Day 26–69**		**Day 69–111**	
	***s*_1_**	**δ_1_ (day^−1^)**	***s*_2_**	**δ_2_ (day^−1^)**	***s*_3_**	**δ_3_ (day^−1^)**	***s*_4_**	**δ_4_ (day^−1^)**
0.05	2.841	−0.278	1.083	−0.215	0.477	0.042	0.603	0.039
0.25	0.569	−0.078	0.217	−0.015	0.095	0.242	0.121	0.240
0.328	0.433	0.000	0.165	0.063	0.073	0.320	0.092	0.318
0.5	0.284	0.172	0.108	0.235	0.048	0.492	0.060	0.490
1	0.142	0.672	0.054	0.735	0.024	0.992	0.030	0.990
5	0.028	4.672	0.011	4.735	0.005	4.992	0.006	4.990
10	0.014	9.672	0.005	9.735	0.002	9.992	0.003	9.990

We fixed the value of replication rate r = r_i_ and calculated segregation probability s_i_ and death rate δ_i_ using linear regressions with Equations (5) and (6). Parameters combinations highlighted by gray boxes were outside physiologically feasible ranges. The minimal constant rate of Mtb replication was 0.328 day^−1^. For a constant replication rate, the segregation probability must have declined 5–7 fold during infection for the model to be consistent with the data.

Finally, we investigated whether the data could be explained by a model in which the rate of cell death is constant during the infection. Because the total number of bacteria continuously increased during the infection (Figure 1A), we first investigated whether these data can be explained with a model in which Mtb death rate is zero. Of note, the original analysis showed non-significant Mtb death rate in 13–26 days post infection but positive death rates in other periods. Interestingly, the model with δ = 0 was not able to explain the data since it required unrealistically high segregation probability during the chronic infection (Table 5 and Figures 3E,F). In fact, the data could only be only explained at the minimal constant rate of Mtb death of δ ≈ 0.05 per day (Table 5). Therefore, our analysis suggested that bacteria must be removed during the infection including the chronic stage of infection. It is interesting to note that the decline in Mtb counts in murine lungs after initial expansion of the bacterial population observed in some (Gallegos et al., 2008; Zhang et al., 2012) but not other (Jung et al., 2005; Gallegos et al., 2011) studies also indicated removal of bacteria. Similarly to the analysis with a constant replication rate, a model with a constant death rate can be consistent with the data but it would require 3–5 fold decline in segregation probability over the course of infection. This decline is smaller than that for a constant replication rate, and yet may be higher than what is expected from *in vitro* experiments. Similarly to the suggestion given above, independent measurements of segregation probability *in vivo* would allow to make a stronger conclusion as to whether there are changes in the rate of Mtb death with time since infection.

**Table 5:** **Data could not be explained assuming immortal bacteria**.

**δ**	**Day 1–13**		**Day 13–26**		**Day 26–69**		**Day 69–111**	
	***s*_1_**	***r*_1_ (day^−1^)**	***s*_2_**	***r*_2_ (day^−1^)**	***s*_3_**	***r*_3_ (day^−1^)**	***s*_4_**	***r*_4_ (day^−1^)**
0	0.433	0.328	0.204	0.265	3.108	0.008	2.920	0.010
0.05	0.376	0.378	0.172	0.315	0.141	0.108	0.499	0.060
0.25	0.246	0.578	0.105	0.515	0.093	0.258	0.116	0.260
0.5	0.172	0.828	0.071	0.765	0.047	0.508	0.059	0.510
1	0.107	1.328	0.043	1.265	0.024	1.008	0.030	1.010
5	0.027	5.328	0.010	5.265	0.005	5.008	0.006	5.010
10	0.014	10.328	0.005	10.265	0.002	10.008	0.003	10.010

We fixed the value of the death rate δ = δ_i_ and calculated segregation probability s_i_ and replication rate δ_i_ using linear regressions with Equations (5) and (6) and experimental data (Figure 1A). Parameters combinations highlighted by gray boxes were are outside physiologically feasible ranges. The minimal constant rate of Mtb death was δ ≈ 0.05 day^−1^. For a constant death rate, the segregation probability must have declined 3–5 fold during infection for the model to be consistent with the data.

### Model predicts that most cells are replicating in the chronic stage of infection

It is generally viewed that a majority of people infected with Mtb will eventually develop a latent infection (Flynn and Chan, 2001; Monack et al., 2004; Gideon and Flynn, 2011). Bacteria in the latent infection are thought to be resistant to drug therapy, and one possibility is that these bacteria are non-replicating *in vivo* but can be “awakened” with appropriate conditions (Zhang, 2004). Our extended model (Equations 1–3), which is based on the previous work (Gill et al., 2009), however, does not include the possibility of latency. Therefore, we changed the model to allow for formation of dormant/quiescent bacterial cells during the infection which are not able to replicate or die but can be recovered (or not) when plated *in vitro* (Figure 2). The model included an additional time-dependent parameter *q*(*t*) describing the probability of formation of a quiescent cell following a division of a replicating cell (see Equations 7–10). Because only two parameters can be estimated in a given time interval from measurements of the total number of bacteria and number of plasmid-bearing bacteria, it was impossible to estimate parameters *r*, δ, *s*, and *q* from these data alone. Therefore, in our analysis we fixed segregation constant to *s* = 0.18 as measured previously in *in vitro* experiments (Gill et al., 2009), fixed quiescence probability to several different values, and estimated remaining parameters *r* and δ. As an additional output of the model, we calculated the fraction of bacteria in the quiescent state out of total bacteria (*%Q* = (*P*_*q*_(111) + *F*_*q*_(111))∕*N*(111)) at 111 days post infection. Percent quiescent bacteria serves as indication of how many bacteria may be quiescent in the chronic phase while there is a decay in the fraction of plasmid-bearing cells in the population, implying bacterial replication (Figure 1B).

**Figure 4:**
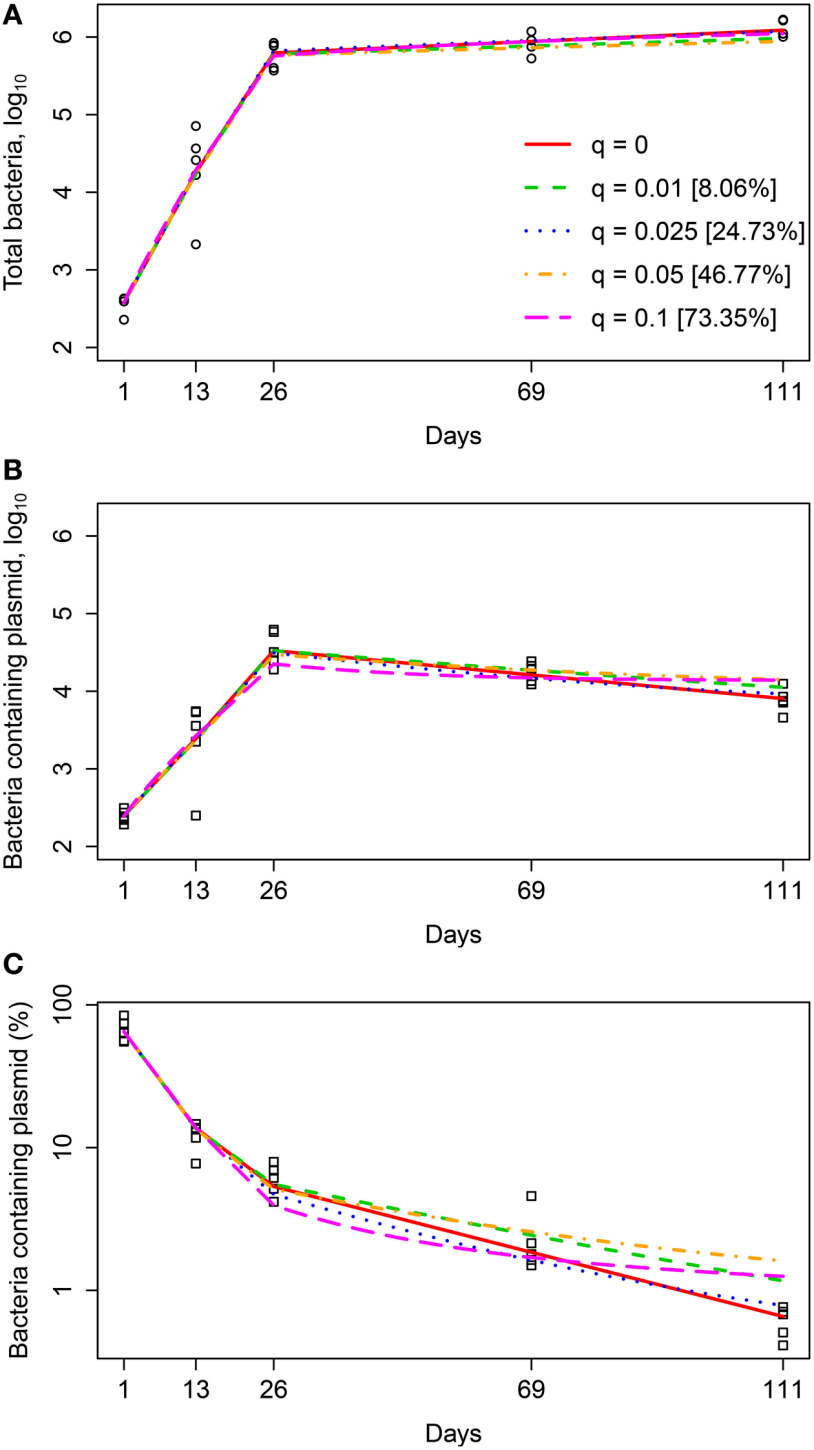
**Quiescence model accurately predicted the dynamics of total number of bacteria (A), number of plasmid-bearing bacteria (B), and the percent of plasmid-bearing cells in the population (C) for a range of quiescence probabilities**. We fitted the mathematical model given in Equations (7–10) to experimental data (Figure 1A) for a fixed value of segregation probability *s* = 0.18 and different fixed values of the quiescence probability *q* and estimated rates of Mtb replication *r* and death δ for four different time periods for our minimal 3*r*∕2δ model (see Equation 4 and Table 2). Estimated model parameters are shown in Table 6 for “Quiescent” model. Markers show experimental data and lines are the model predictions. Model fits for several different values of the quiescence rate are shown and values in square brackets are the percent of bacteria at day 111 which are in the quiescent state. Fits were of a similar quality for models in which bacteria become quiescent only in the chronic phase of infection [*q*(*t*) = 0 if *t* < 26 and *q*(*t*) = *q*, otherwise], when quiescent bacteria are not counted during *in vitro* plating, or when all different values are allowed for replication and death rates for the four time intervals (see Table 6 and results not shown).

Interestingly, a range of quiescence probabilities allowed for reasonably good fits of the data with relatively low AIC scores with the values of *q* ≤ 0.05 resulting in fits of identical quality (Figure 4 and Table 6). As expected higher probabilities of quiescence resulted in higher rates of replication and death (Table 6). Surprisingly, independently of the time when generation of quiescent cells started, the model predicted that a large fraction of bacteria at the end of the experiment (day 111) could be in non-replicating, quiescent state. In fact, ~40% could be in non-replicative state, while other bacteria replicated and died at biologically reasonable rates in chronic infection and the model fits are also acceptable from a statistical perspective (Figure 4). Thus, our analysis suggests that a large fraction of quiescent, non-replicative bacteria is not inconsistent with the data of Gill et al. (2009). If quiescent bacteria cannot be recovered by plating *in vitro* even larger percents of quiescent bacteria would be consistent with the data (Table 6). If all bacteria can be plated *in vitro* our results also suggest that at least 60% of bacteria must be in the state of active replication during the chronic phase of infection at the end of the experiment (day 111). This conclusion further challenges the widespread belief of limited Mtb replication during the chronic phase of infection.

**Table 6:** **Analysis predicted a high fraction of quiescent bacteria in the chronic phase of infection**.

	***q***	***r*_1_**	***r*_2_**	***r*_3_**	**δ_1_**	**δ_2_**	**AIC**	**SSR**	**%*Q***
**Quiescent**	0	0.73	0.40	0.14	0.40	0.13	−124.72	3.29	0
	0.01	0.72	0.42	0.15	0.39	0.14	−124.35	3.31	8.06
	0.025	0.72	0.45	0.17	0.38	0.16	−123.50	3.37	24.73
	0.05	0.75	0.48	0.19	0.41	0.18	−121.22	3.52	46.77
	0.1	0.83	0.51	0.20	0.45	0.18	−117.28	3.82	73.35
**Quiescent Late**	0.01	0.72	0.41	0.15	0.39	0.14	−124.51	3.31	8.66
	0.025	0.72	0.42	0.16	0.38	0.15	−124.0	3.34	21.83
	0.05	0.73	0.44	0.18	0.41	0.17	−122.72	3.42	42.67
	0.1	0.78	0.46	0.20	0.45	0.19	−119.77	3.63	72.69
**Quiescent bacteria not counted**	0.05	0.72	0.42	0.14	0.36	0.12	−124.64	3.29	31.55
	0.1	0.71	0.43	0.13	0.32	0.11	−124.54	3.20	47.89
	0.2	0.69	0.47	0.13	0.23	0.10	−124.27	3.32	64.59

We fitted the quiescence model (Equations 7–10) to experimental data (Figure 1A) with s = 0.18 and different values of q (indicated in the table) and estimated Mtb replication (r, day^−1^) and death (δ, day^−1^) rates for different time periods (see Equation 4) assuming that there are 3 replication and 2 death rates (see Table 2). In different fits we assumed that quiescent cells were formed during the whole infection [“Quiescent,” q(t) = q], quiescent cells were formed only in chronic phase of infection [“Quiescent Late,” q(t) = 0 if t < 26 and q(t) = q, otherwise], quiescent cells were formed during the whole infection but could not be counted during standard plating procedure [“Quiescent bacteria not counted,” q(t) = q].

### Alternative models

The original study of Gill et al. (2009) and our analysis so far assumed that the main reason for the loss of plasmid-bearing cells in the Mtb population was a high probability of plasmid loss at cell division (so-called segregational instability of plasmids). However, a lower replication rate of plasmid-bearing bacterial cells has been shown to contribute to the accumulation of plasmid-free cells in some conditions (Zünd and Lebek, 1980; Bentley et al., 1990; Ganusov and Brilkov, 2002). Reanalysis of the data on the dynamics of fraction of plasmid-bearing Mtb cells *in vitro* (Gill et al., 2009) with a model that assumes both fitness cost to carrying a plasmid (*c* > 0) and segregational instability (*s* > 0) suggested that a moderate segregation probability (*s* ≈ 0.04) and substantial fitness cost (*c* ≈ 0.16) are consistent with experimental data (results not shown). Therefore we fitted a mathematical model (Equations 11 and 12) to experimental data on the dynamics of Mtb in murine lungs (Figure 1A) and estimated Mtb replication and death rates for four time periods with *s* = 0.04 and *c* = 0.16. Because in this model the decline in the frequency of plasmid-bearing cells occurs due to cell division, model fitting suggested a decline in the rates of Mtb replication and death with time since infection, and further analysis showed that three replication and two death rates are needed to accurately explain the data [results not shown and *F*-test for nested models, *F*_(3, 40)_ = 0.06, *p* = 0.98]. While qualitatively the results were independent of the exact mechanism of plasmid loss in the population, the model with a high fitness cost of the plasmid led to higher estimates of Mtb replication and death rates in early infection. Specifically, we found estimates *r*_1_ = 1.2/day and δ_1_ = 0.79/day which are much higher than those in the model in which plasmid loss is driven by high segregation probability (e.g., Table 2). Because estimates of the Mtb replication and death rates in the chronic phase were similar in two models (results not show), this analysis suggests that the reduction in Mtb replication rate over the course of 111 days in mice may be even more substantial than previous analysis suggested (Gill et al., 2009).

Because in our constrained (“3*r*∕2δ”) model we observed substantial changes in the rate of Mtb replication and smaller changes in the rate of Mtb death we investigated if a more mechanistic mathematical model, based on logistic growth, was able to describe experimental data. Therefore, we formulated a model in which replication of bacteria is described logistically (see Equations 15 and 16), plasmid segregation probability is constant (*s* = 0.18), and bacteria are removed at a constant rate δ. Surprisingly, this model was able to accurately describe the change in the total number and number of plasmid-bearing bacteria over the course of infection [results not shown and *SSR* = 3.55, *F*_(5, 40)_ = 0.70 and *p* = 0.63 for lack of fit test]. The AIC score was similar for the logistic model and our minimal 3*r*∕2δ model suggesting that a model in which bacteria died at a constant rate during the infection might be also consistent with the data. However, a closer inspection of the fit of the logistic model to data suggested that the model was not able to accurately predict a slow rise in the Mtb counts in the lung in the chronic infection (see Figure 1A) and decline in percent of plasmid-bearing cells during the first 3 weeks of infection (results not shown). The latter observation suggests that the type of experimental data used for fitting models may influence the model selection process. Indeed, additional analysis suggested that our extended model assuming three replication and one death rates could well describe experimental data on the dynamics of total number of bacteria and total number of plasmid-bearing bacteria judged by the lack of fit test or *F*-test for nested models (results not shown). Yet, similarly to the logistic model, the 3*r*∕1δ model could not accurately predict the decline in the percent of plasmid-bearing cells in the population. Thus, future studies utilizing Gill et al. (2009) data should focus on describing the dynamics of the total number of bacteria and the percent of plasmid-bearing cells in the population using, for example, general likelihood or weighted least squares methods (Ganusov et al., 2010).

## 4. Discussion

While Mtb is thought to cause a persistent infection in many exposed individuals, the state of bacteria during the latent infection is not fully understood. Although there are many differences in the disease caused by Mtb in humans and mice, mice remain one of the main animal models to study Mtb pathogenesis. Recent work by utilizing a novel Mtb strain and a simple mathematical model suggested that during a chronic phase of Mtb infection of mice, bacteria replicate at substantial rates (Gill et al., 2009). Here we extend this pioneering study to determine whether previous conclusions are robust to changes in the model assumptions.

The first assumption we challenged was that the probability of plasmid segregation is constant during the infection. It is known that environmental conditions can influence the segregational stability of plasmids, as well as contribute to plasmid deamplification (Smith and Bidochka, 1998). The metabolic stress of maintaining plasmids in unfavorable conditions can be alleviated through changes in probability of plasmid loss during division (Smith and Bidochka, 1998). Studies conducted on *E. coli* transformed with recombinant plasmids and kept in continuous culture have shown that the probability of plasmid loss is not constant and varies between different growth rates (Wouters et al., 1980; Popova et al., 1992; Mosrati et al., 1993; Smith and Bidochka, 1998; Ganusov and Brilkov, 2002). In particular, Mosrati et al. (1993) showed that the probability of plasmid loss in this particular case was not constant, and was positively correlated with the rate of growth of plasmid-bearing bacteria. Allowing the probability of plasmid segregation to change over the course of Mtb infection led to several interesting results. First, our analysis suggested that while in our data the total number of bacteria always increased over the course of infection, the data could not be explained assuming immortal bacteria. Second, allowing segregation probability to vary influenced the actual estimates of the Mtb replication and death rates. Third, relatively large changes in segregation probability were needed to explain experimental data with constant replication or death rates, suggesting that previously found decline in both rates over the course of infection is likely a robust result (unless the segregation probability declines several fold with time since infection). Fourth and finally, our analysis puts limits on the constant rates of Mtb replication (*r*_*min*_ = 0.328 day^−1^), death (δ_*min*_ ≈ 0.05 day^−1^), and segregation probability (*s*_*max*_ = 0.204) for the model to be consistent with the data.

The second assumption was that all bacteria during the infection were capable of replicating. There is clear evidence that over the course of Mtb infection of mice, there are changes in Mtb metabolism which could potentially result in dormancy or non-replicative state (Barry et al., 2009; Boon and Dick, 2012). In particular, Schnappinger et al. (2003) measured transcriptional responses of Mtb upon phagocytosis by macrophages. Transcription profile showed expression of genes which correspond to adaptations to a nitrosative, oxidative, and hypoxic environment. Genes responsible for cell envelope repair were also actively expressed. This shows evidence of a harsh environment within the phagosome; adaptations to these conditions have been reported to be associated with a dormancy-related phenotype in Mtb. Genes responsible for fatty-acid metabolism were also upregulated in this study, indicating that the primary carbon source within the phagosome are fatty acids. *In vitro* studies have also shown that when Mtb is grown in the presence of long-chain fatty acids as the primary carbon source, it enters a state genetically comparable to dormancy (Rodríguez et al., 2014).

Extending the previous model to allow for formation of quiescent, non-replicating bacteria led to two interesting predictions. First, a range of quiescence probabilities was consistent with experimental data but at higher values the model fits predicted a large increase in replication and death rates in the chronic infection for several versions of the model (Table 6). Such an increase in the replication rate is unlikely to be true biologically (Barry et al., 2009). Second, for biologically reasonable parameters about 25–40% of bacteria recovered at the end of experiments (day 111 post infection) could be in dormant, non-replicative stage. Thus, these experimental data are compatible with the notion that many bacteria during chronic infection are non-replicating even though there is a continuous loss of plasmid-bearing cells in the population (Figure 1B). Our analysis thus put a limit on the fraction of quiescent bacteria which are still expected to be the minority of the population. Our results also suggested that during chronic phase of infection the majority of mycobacterial cells in the lung must be replicating.

In our analysis we assumed that these quiescent bacteria were unable to die. This was due to the idea that Mtb death occurs during bacterial replication. We also tested models which assumed that quiescent bacteria would die at the same rate as non-quiescent bacteria and it was found that a model with even higher rates of quiescence is able to explain the data, although that model predicted a smaller fraction of quiescent bacteria at the end of experiments (Table S1 in Supplemental Information).

Additional experiments will be needed to determine the size of the fraction of non-replicating bacteria during the chronic infection. One set of such experiments could involve treatment of mice, infected with this Mtb strain, with antibiotics. For example, isoniazid is currently believed to kill rapidly dividing mycobacteria while pyrazinamide or rifampin also act on slowly growing or nonreplicating bacteria (Mitchison, 2000; Horsburgh et al., 2015). Treating Mtb-infected mice with different antibiotics and recording the kinetics of loss of plasmid-bearing cells would allow for an independent test of the fraction of rapidly replicating bacteria in the chronic phase of infection (given that our current understanding of how these drugs work *in vivo* in mice is correct, Horsburgh et al., 2015).

**Figure 5:**
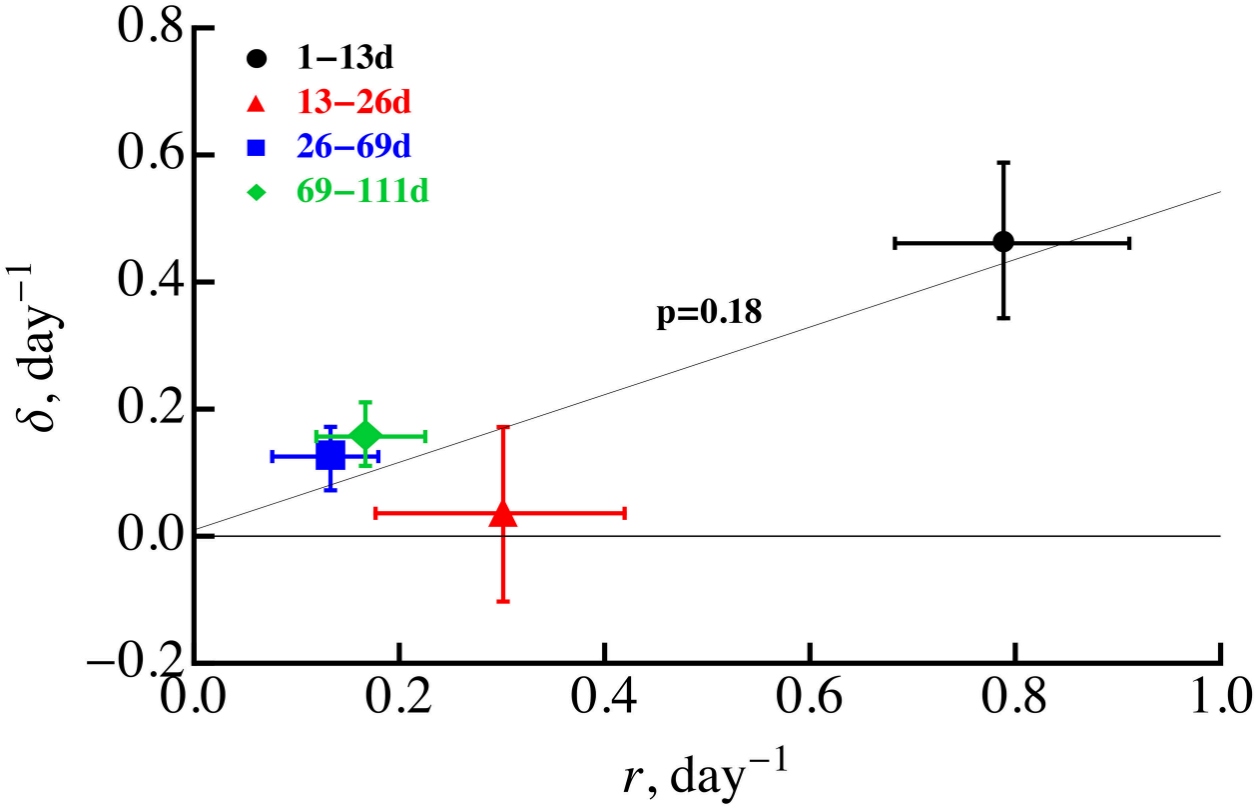
**Absence of significant correlation between the rate of Mtb replication and rate of Mtb death during the infection**. We estimated the rates of Mtb replication (*r*) and death (δ) for four time periods using linear regressions (Equations 5 and 6) and letting *s* = 0.18. These estimates are identical to those given in Gill et al. (2009). Statistical analysis of the correlation was performed using Spearman rank correlation with *p*-value indicated on the graph. Error bars denote confidence intervals for parameter estimates obtained by bootstrapping data from individual animals 10^4^ times. The regression line was drawn for illustrative purposes.

Our analysis still supports a previous conclusion that both Mtb replication and death rate are likely to decline during the infection (Gill et al., 2009) unless there are large changes in the probability of plasmid loss. Statistical tests suggested that for a constant probability of plasmid segregation (*s* = 0.18), replication rate declines during first 2 months post infection while death rate only declines in the first 3 weeks. Decline in the replication rate over the course of infection may be expected as bacteria exhaust host's resources (e.g., macrophages) or due to formation of granulomas which may limit access of bacteria to nutrients such as oxygen. However, the decline in the death rate over the course of infection was unexpected since 3–4 weeks post-infected, Mtb-specific T cells arrive to the lung (Jung et al., 2005) and are expected to enhance macrophage-mediated killing of bacteria. If there was a strong correlation between the rate of bacterial replication and cell death, for example if cells are most susceptible to die during cell division, we would expect to see a decline in the rate of cell death (because the rate of cell replication declines). Indeed, several previous studies found that some bacteria are very susceptible to antibiotics when they rapidly divide providing indirect support for this hypothesis (Tuomanen et al., 1986; Evans et al., 1990; Gilbert et al., 1990) (but see Raffetseder et al., 2014). However, we found no significant correlation between Mtb replication and death rates for 4 time periods, in part due to unexpectedly low death death rate in 13–26 days post infection (Figure 5). This non-significant correlation was further confirmed by the inability of the ODE-based model (Equations 1 and 2) assuming δ_*i*_ = α*r*_*i*_ with α = *const* to accurately explain experimental data [results not shown and *F*_(2, 40)_ = 8.46 and *p* < 0.001 in lack of fit test]. Thus, further studies are needed to explain why the Mtb death rate declined during the first 3 weeks post infection.

Although our extensions of the mathematical model of Gill et al. (2009) were relatively simple, they still ignored several additional mechanisms which can also impact estimates of the Mtb replication and death rates. For example, the pBP10 plasmid may bear a fitness cost and thus could reduce the rate of replication of plasmid-bearing cells. Gill et al. (2009) showed that presence of a small fitness cost will lead to higher estimates for the replication and death rates of bacteria. We extended their result by fitting an alternative model, in which plasmid loss probability is small and fitness cost is large, to experimental data. The general result that both replication and death rates decline during the infection remained robust but numerically indeed, high fitness cost led to higher estimates of the Mtb replication and death rates during the first 2 weeks of infection. It should be noted, however, that given strikingly exponential increase in the total number of bacterial cells and decline in percent of plasmid-bearing cells in *in vitro* experiments (Gill et al., 2009), it is unlikely that carriage of plasmid bears high fitness cost. Previous analyses illustrated that if fitness cost of carrying the plasmid is large, population size and percent of plasmid-bearing cells would change according to a bi-exponential function, and not single exponential (Ganusov and Brilkov, 2002, and results not shown).

Another assumption of our and previous analyses was that plasmids are lost from the cell only following cell division. While it is not very likely, plasmid could be also lost from a cell due to degradation process, activated in the chronic infection. Additional data are needed to confirm stability of the plasmid in harsh, *in vivo*-like conditions.

All of our analysis involved the data on the dynamics of the total number and the number of plasmid-bearing Mtb cells in the lung (Figure 1A). A closer inspection of the data revealed one outlier mouse which had significantly lower Mtb counts in the lung at day 13 post infection. While we have no biological reasons to exclude this specific data point from our main analyses, we nevertheless repeated most of our analyses excluding that outlier mouse from the data. Although excluding the outlier did affect estimates of several parameters, none of the conclusions were affected (results not shown). Because we also performed simulations to evaluate confidence intervals for parameter estimates, we believe that major conclusions reached in our paper were robust to that specific peculiarity in the data.

A series of detailed mathematical models of the within-host dynamics of Mtb has been developed in the last 10 years (Gammack et al., 2004; Marino and Kirschner, 2004; Kirschner and Marino, 2005; Fallahi-Sichani et al., 2011; Marino et al., 2011; Pienaar et al., 2015). Such models took into account the complexity of the Mtb replication in macrophages and Mtb-specific immune response in the lymph nodes and lung; however, very limited *in vivo* experimental data were used to calibrate such models. Most of parameters of such models (e.g., Marino and Kirschner, 2004; Fallahi-Sichani et al., 2011) were estimated from indirect experimental data on Mtb growth *in vitro* or *in vitro* and *in vivo* immune responses to model antigens or other pathogens. Many other parameters were guessed to constrain model dynamics. We do not know if one version of such models would be able to explain Gill et al. (2009) data but given the complexity of such detailed models and the number of free parameters, it is very likely. However, model overfitting of the data should not be viewed as model confirmation (Oreskes et al., 1994). As Enrico Fermi, a Nobel prize laureate, once said: “with four parameters I can fit an elephant, and with five, I can make him wiggle his trunk” (Mayer et al., 2010; Ditlev et al., 2013). There is a need for relative simple mathematical models of within-host Mtb dynamics whose complexity is driven not by the biological complexity of the phenomenon (which can be explored *ad infinitum*), but by the inability of simpler models to accurately describe *in vivo* data. This is in line with “strong inference” in science (Platt, 1964). In particular, it remains to be determined if simple, so-called standard models for the dynamics of intracellular pathogens (Perelson, 2002), are able to explain experimental data of Gill et al. (2009). Given that logistic model is able to only partially explain these data it is possible that more complex mechanistic models are indeed needed to accurately describe Gill et al. (2009) data (e.g., Figure 1B).

Since our analysis was done for the data coming from Mtb infection of mice, relevance of our findings to Mtb infection of humans remains unclear. Clearly, many aspects of TB pathogenesis are different between mice and humans, and more recent studies suggested that Mtb dynamics in monkeys may be more representative of what happens in humans (Scanga and Flynn, 2014; Flynn et al., 2015). Such conjecture can be debated given our limited understanding of human TB. Detailed data on Mtb dynamics in monkeys, similar in quality and amount to the work of Gill et al. (2009) are not yet publicly available for analysis, so application of our modeling approach to Mtb infection of monkeys will be only possible when similar data are generated and made available. For example, infection of monkeys with this Mtb strain may provide information on the birth and death processes regulating the number of bacteria found in individual granulomas (Lin et al., 2014). At this point our analysis provided quantitative estimates on how Mtb may behave in mice and future work in monkeys and humans will be needed to evaluate relevance of these findings to human TB.

One of the main conclusions of our work is that even for a quite extensive set of experimental data and relatively simple mathematical models, estimates of the model parameters are not always robust to the changes in model assumptions and some of our conclusions challenge previous results. Furthermore, alternative models (e.g., a model the dormant/quiescent state) can also explain the data with similar quality. Our analysis thus suggests that to constrain model predictions and to discriminate between alternative models additional measurements are needed. As we mentioned earlier, several different mechanistic models for the Mtb replication in mice and humans have been constructed (e.g., see review Kirschner and Marino, 2005); however, which mechanisms of such complex models are truly needed to explain major qualitative and quantitative features of Mtb infection in mice remains undefined. Experimental data such as those of Gill et al. (2009) as well as other studies utilizing plasmid pBP10 could be a valuable source for testing and rejecting different mechanistic models of Mtb replication in mice and possibly in monkeys.

## Author contribution

Designed the study: SE and VVG. Performed simulations and model fitting: MMM, NK, WGH, VVG. Analyzed results: MMM, NK, WGH, SE, VVG. Wrote the paper: MMM, NK, WGH, SE, VVG.

### Conflict of interest statement

The authors declare that the research was conducted in the absence of any commercial or financial relationships that could be construed as a potential conflict of interest.
