# Supplementary material for: Quantifying Limits on Replication, Death, and Quiescence of Mycobacterium tuberculosis in Mice
Source: Front Microbiol. 2016 Jun 14;7:862. doi: 10.3389/fmicb.2016.00862 (PMC4906525; doi:10.3389/fmicb.2016.00862)
Supplement: Supplementary file 1 [file Table1.pdf]

Supplemental Material

q	r_1	r_2	r_3	δ_1	δ_2	AIC	SSR
0	0.722	0.402	0.137	0.400	0.129	-124.72	3.28
0.01	0.750	0.402	0.130	0.421	0.122	-124.61	3.60
0.025	0.735	0.399	0.131	0.395	0.120	-124.57	3.29
0.05	0.740	0.402	0.128	0.383	0.114	-124.44	3.31
0.1	0.756	0.414	0.113	0.375	0.095	-123.34	3.60
0.2	0.798	0.482	0.096	0.341	0.076	-120.03	3.81
0.3	0.859	0.533	0.141	0.332	0.085	-111.77	4.26

Table S1: Higher probability of quiescence was consistent with experimental data when both replicating and quiescent bacteria die during infection. We extended the mathematical model with quiescent bacteria (eqns. (7)–(10)) to allow all bacteria to die at a constant rate δ which was dependent on the time period. We fixed the rate of quiescence to values shown, let $s = 0.18$ and estimated rates of Mtb replication and death for our minimal $3r/2\delta$ model. In the table, rates r and δ are in day^{-1} units. Note that at $q = 0.1$ the model describes the data with reasonable quality as judged by AIC. Analysis of the mathematical model shows that over a sufficiently long time period, the fraction of quiescent bacteria in the population is equal to q (results not shown). Thus, allowing for the removal of quiescent bacteria predicts even higher fraction of replicating cells in the chronic phase of infection (or a smaller fraction of quiescent bacteria).
